# Botulinum Toxin Injections to Manage Sequelae of Peripheral Facial Palsy

**DOI:** 10.3390/toxins16030161

**Published:** 2024-03-20

**Authors:** Fabienne Carré, Jérémy Amar, Frédéric Tankéré, Claire Foirest

**Affiliations:** ENT Department, Pitié-Salpêtrière’s Hospital, Sorbonne Université, 7-83 Boulevard de l’Hôpital, 75013 Paris, France

**Keywords:** botulinum toxin, facial paresis, synkinesis, sequelae, facial asymmetry, post paralytic facial spasm

## Abstract

Long-standing facial palsy sequelae cause functional, aesthetic, and psychological problems in patients. Botulinum toxin is an effective way to manage them, but no standardized recommendations exist. Through this non-systematic review, we aimed to guide any practitioner willing to master the ins and outs of this activity. We reviewed the existing literature and completed, with our experience as a reference center, different strategies of botulinum toxin injections used in facial palsy patients, including history, physiopathology, facial analysis, dosages, injection sites, and techniques, as well as time intervals between injections. The reader will find all the theorical information needed to best guide injections according to the patient’s complaint, which is the most important information to consider.

## 1. Introduction

Idiopathic peripheral facial palsy is a common condition, with an annual incidence of 14 to 25/100,000 people [1]. In 30% of cases, patients possess sequelae such as hemifacial spasm, synkinesias, and spasticity [2,3]. Patients with non-idiopathic facial paralysis, particularly secondary to surgery, may also possess this type of complication [4,5].

Prognostic factors for these sequelae include a grade higher than II in the House and Brackmann classification, a score lower than 85% in the Sunnybrook grading system (SFGS), and treatment after 4 days post-paralysis [6].

These sequelae impact the patient psychosocially, aesthetically, and functionally [7]. Their management may involve muscle and speech rehabilitation, botulinum toxins, or surgical techniques [2].

Medical treatment of the aftereffects of facial paralysis using botulinum toxin injections is gaining ground. Injections target the neuromuscular synapses of the muscles responsible for spasms, synkinesis, and hyperkinesis. When the facial paralysis is flaccid, an injection of toxin in the contralateral region can symmetrize the face or treat any hyperactivity [8,9]. It can also improve mobility on the paralyzed side [10]. In this way, botulinum injections increase the quality of life for patients [11]. The side effects are minor and reversible over time.

In recent years, there has been an explosion in the use of botulinum toxin to improve objective and subjective aftereffects. The number of doctors injecting botulinum toxin has also increased exponentially to meet this demand.

However, unlike cosmetic injections, there are currently no best-practice recommendations for botulinum toxin injections in patients with sequelae of facial paralysis [12]. Each injecting physician evaluates and conducts an injection protocol based on his or her own experience and the patient’s complaints.

The aim of this work is to clarify and guide the use of botulinum toxin injections in the management of patients with sequelae of facial paralysis. To do this, we draw on the practical experience of the ENT department in a tertiary care center and the national reference center for facial palsy patients, sharing our experience and practice as well as analysing the literature. We selected from the Pubmed database all the relevant articles dealing with the management of botulinum toxin injection of patients with sequelae of facial paralysis. Keywords were “botulinum toxin”, “facial palsy” or “facial paralysis”, “sequalae”, “synkinesis”, “spasm”, and “facial asymmetry”. Clinical cases, cohorts, and literature reviews were included. Non-English language items were excluded.

## 2. History and Physiology of Botulinum Toxin

Botulinum toxin is a neurotoxin secreted by a bacterium, *Clostridium botulinum*, which is responsible for botulism. Its mechanism of action is well known [13]. By inhibiting acetylcholine release at the neuromuscular junction synapse, botulinum toxin causes flaccid muscle paralysis. It was first used therapeutically in 1973 to treat strabismus [14]. The indications for botulinum toxin subsequently extended to a wide range of pathologies, and the first specific publications on its use in the aftereffects of facial paralysis date from the late 1980s [15]. In recent years, botulinum toxin has played an increasingly important role in the management of the sequelae of peripheral facial paralysis [8,16].

By studying the topographical distribution of the neuromuscular units of the facial musculature, it has been possible to specify the various botulinum toxin-injection points in order to target the desired effects of the toxin and deliver an optimal dose [17,18].

In particular, it treats spasms, co-contractions, and synkinesis, as well as sequelae of facial asymmetry on the paralyzed side if it is spastic. It can also reduce the amplitude of movement on the healthy side and even treat contralateral hyperactivity.

## 3. Sequelae of Peripheral Facial Paralysis

### 3.1. Clinical Presentations

There are typically two clinical profiles in patients with unilateral peripheral easy paralysis, whatever the etiology: flaccid paralysis and spastic paralysis. In each case, it is important to describe the paralyzed side and the healthy side, which will be witness to specific signs.

#### 3.1.1. Flaccid Paralysis

The paralyzed side shows hypotonia of the hemifacial region, with the following classic features: obliteration of forehead wrinkles, ptosis of the eyebrow, lagophthalmos, and ectropion defining a scleral show and potentially leading to exposure keratitis, obliteration of the nasolabial fold, and the fall of the labial commissure, resulting in labial incompetence, difficulties with chewing, articulation, speech, or biting.

On the healthy side, compensatory muscular hypertonicity and the hypercontraction of the facial muscles may appear, either statically or dynamically, classically resulting in an elevation of the eyebrow and reduction of the palpebral cleft, more pronounced wrinkles, attraction of the labial commissure, and bitterness folds.

#### 3.1.2. Spastic Paralysis

The paralyzed side is affected by the presence of muscle spasticity, synkinesis, co-contractions, and spasms. On the healthy side, hyperactivity may be present but is often less marked than in flaccid paralysis.

Facial asymmetry increases with age, with wrinkles often more pronounced on the healthy side. The evolution over time of synkinesia can also be responsible for worsening asymmetry.

### 3.2. Type of Motor Sequelae

Co-contractions or synkinesis are involuntary concomitant muscle contractions of a muscle or muscle group of the face different from the one the patient wants to contract. Their pathophysiology is complex and may result from the complex regeneration of facial nerve fibers responsible for aberrant reinnervation [19,20,21]. They classically develop during the period of neuronal repair 3-to-6 months after nerve trauma or the onset of facial paralysis [16,22]. Synkinesis is particularly noticeable during spontaneous facial movements, especially during emotional expressions such as smiling. Oculo-oral and oro-ocular synkinesis are the most frequently encountered. However, all possible types of synkinesis should be investigated for different facial movements: eye–chin, eye–mouth–platysma, eye-front, and zygomatic–depressor anguli ori [3,23].

The prevalence of synkinesis after peripheral facial paralysis ranges from 15 to 56%, depending on the series [1,3,23,24]. They are the most frequent complaint of patients with sequelae of peripheral facial paralysis, a source of chronic physical and mental pain [25]. Reactive contracture of the healthy side is also responsible for facial asymmetry, which impairs the quality of life in these patients [26,27].

Spasms are involuntary, spontaneous contractions of the facial muscles on the paralyzed side. They can be brief and rhythmic or sustained and irreducible. Their pathophysiology involves Wallerian axon degeneration [28]. Facial motor dysfunction can be spontaneous or triggered by any type of facial movement, whether automatic or emotional. Classically, the spastic muscle is also involved in synkinesis [29]. Clinically, these spasms may partly mimic essential hemifacial spasms, but neurophysiopathology is quite distinct.

Hyperkinesia increases static or dynamic asymmetry of the face, leading to significant functional and aesthetic problems for the patient, such as a pronounced nasolabial fold, mouth deviation, and a narrower palpebral fissure than on the contralateral side. This mechanism results from the hyperactivity of the facial muscles on the non-paralyzed side against the weak antagonism of the muscles on the paralyzed side. This mechanism also increases wrinkles on the non-paralyzed side, particularly in the forehead and glabella, with eyebrow asymmetry and deviation of the nose and mouth on the non-paralyzed but hyperactive side [3,30].

Myokymia is an involuntary, localized quivering of a few muscle fibers that is not strong enough to generate movement. Classically, patients with sequelae of facial paralysis may exhibit involuntary eyelid tremor [28,31].

Crocodile tear syndrome, or gusto–lacrymal syndrome, is thought to reflect a regrowth of parasympathetic vegetative fibers of the facial nerve innervating the parotid gland, leading to aberrant reinnervation of the lacrimal and sweat glands via the auriculotemporal nerve [32].

### 3.3. Clinical Evaluation Scales

The House and Brackmann assessment scale evaluates facial motricity at rest and in movement but does not consider synkinesis [33]. Thus, a patient with facial function assessed as normal at rest may present very disabling synkinesis. He would then be assessed as Grade III according to the House and Brackmann classification.

Various scales for evaluating synkinesis can be used to describe them and monitor them over time, particularly during speech therapy but also after botulinum toxin treatment. The Sunnybrook Facial Grading System (SFGS) is one such simple-to-use, reproducible scale, which, thanks to a composite score that includes synkinesis and enables the function of the paralyzed hemiface to be rated out of 100 [34,35]. The Synkinesis Assessment Questionnaire (SAQ) is a specific synkinesis assessment questionnaire based on nine questions rated from 1 to 5 assessing synkinesis on the paralyzed side [36,37].

## 4. Toxin Botulinum in Patients with Sequelae of Peripheral Facial Paralysis

### 4.1. Indications

For most patients, treating involuntary co-contractions and muscle spasticity is more important than achieving perfect facial symmetry [7,26]. The use of botulinum toxin achieves these first objectives while creating a therapeutic window for neuromuscular and oro-myofacial re-education for the targeted management of one muscle or its coordination with others without the hindrance of co-contraction or hyperactivity [38,39].

#### 4.1.1. Treatment of Hyperactivity Contralateral to Paralysis and Symmetrization

Although facial asymmetry is initially due to muscular weakness on the paralyzed side, which attenuates the nasolabial fold, it lowers the angle of the mouth, lowers the eyebrow, and erases forehead wrinkles, while muscular compensation on the healthy side increases wrinkles and furrows. Treatment of this contralateral hyperactivity helps to restore a degree of facial symmetry at rest and during movement [9]. The indication can be validated as soon as hyperactivity is identified to facilitate speech therapy. Facial asymmetry is improved for the duration of the toxin’s efficacy but continues beyond its onset of action, with studies showing persistent improvement in facial asymmetry at 6 months post-injection [40] or even permanently [10].

#### 4.1.2. Treatment of Synkinesis

Botulinum toxin reduces or even eliminates the contractibility of the involuntarily co-contracted muscle or muscle group. Botulinum Toxin A (BTX-A) is frequently used as a safe and effective treatment [22,24,26,29,41,42]. Injecting the muscle with synkinesis at the right dose blocks this involuntary contraction while allowing the muscle to maintain its initial motor function.

#### 4.1.3. Treatment of Spasms

In the case of brief, rhythmic contractions of the muscle on the paralysed side, we need to identify the trigger zone responsible for the muscle discharge. Identifying this zone is a technical task. Ideally, small but more concentrated volumes should be injected to target this area and treat it while minimizing the risk of diffusion [30].

In the case of a permanent contracture, care must be taken to avoid the risk of paralyzing the affected muscle by lifting the contracture. This is particularly true of the zygomatic spasm, which often involves a residual paretic component in voluntary movements.

#### 4.1.4. Recovery Aid for the Affected Side

In the case of residual paresis, some studies have found a benefit from injecting toxin into the opposite hyperactive muscle [5,10,30,43]. Injection of botulinum toxin into the healthy side may benefit the paralyzed side, with an improvement in paralysis [10]. The mechanisms evoked are the facilitation of muscle work or nerve regeneration, as well as brain plasticity [44].

### 4.2. Toxin Type and Dilution

All therapeutic types of botulinum toxin can be used to treat the aftereffects of facial paralysis. Three types of Type A botulinum toxin are indicated for therapeutic use:

- Botox—Onabotulinum toxin, Allergan Inc., Irvine, CA, USA
- Xeomin—Incobotulinum toxin, Merz Pharmaceuticals Frankfurt am Main, Germany
- Dysport—Abobotulinum toxin, Ipsen, Paris, France

These three formulations have the same mode of action. Doses and dilutions vary according to the commercial specialties [16]. Botox and Xeomin have comparable effects with a 1:1 ratio, and their equivalence is no longer in doubt [45,46,47]. In the case of Dysport, a conversion ratio of 1:3 provides equivalence with Botox and Xeomin [48,49].

The doses and dilutions classically used are shown in Table 1 [30].

### 4.3. Pre-Injection Clinical Assessment

The initial patient assessment and understanding of the history of facial paralysis and its evolution over time is imperative. During this consultation, precautions should be taken and the usual contraindications to botulinum toxin injection should be investigated, including pregnancy and breastfeeding, as well as the existence of neuromuscular diseases [12,50].

Few centers offer an assessment and management of these sequelae of facial paralysis. The small number of trained practitioners, combined with a lack of awareness of this type of rehabilitation, means that patients have to wait a long time for treatment.

It is customary not to inject botulinum toxin before 6 months of evolution of facial paralysis due to the possible risk of increased synkinesis [16,51]. However, some authors recommend an early injection on the healthy side to combat hyperkinesia and symmetrize the face at an early stage [52,53]. Akulov et al. have shown, in the management of facial paralysis sequelae of neurosurgery, that an injection of botulinum toxin in the acute phase of the onset of facial paralysis has an early clinical effect on facial symmetrization and may reduce complications such as synkinesis and contractures in the long term [4].

A good understanding of the anatomy of facial muscles and their action on facial expressions is essential to determine the botulinum toxin-injection strategy, the muscles targeted, and the optimal doses [15,27]. 

Muscle tone at rest is assessed as a whole, then each muscle is assessed individually at rest and at maximum contraction. Typically, the patient is assessed at rest, eyebrow elevation, full palpebral closure without and with forcing, nose wrinkling, smiling, labial protrusion, pouting, whistling, and lowering of the lower lip. These movements can also be used to search for synkinesis triggers.

An analysis of facial wrinkles, aberrant contractures, and residual hypotonia is a key element in understanding and treating the muscles involved in facial paralysis sequelae. An injection plan is then drawn up and diagrammed. Photos and videos allow us to better analyze facial dynamics and serve as a reference for patient follow-up after injections.

### 4.4. Practical Application: Injection Sites

Knowledge of the anatomy and function of facial muscles is essential for understanding botulinum toxin-injection sites and protocols. Injection techniques are essential to minimize side effects and optimize treatment efficacy. The precise area of injection, as well as the depth and angle of the needle, is essential.

Some centers use facial electromyography to identify non-subcutaneous muscles and inject them [54]. Electromyography also provides immediate feedback on the muscle responsible for synkinesis and enables it to be treated. It can also be used to objectivize the degree of hypertonia and residual paresis of a selected muscle in comparison with the contralateral muscle.

#### 4.4.1. Upper Third of Face

Treatment of the upper third of the face involves maintaining a natural appearance and balance between the paretic/synkinesis side and the affected side, as well as between the muscles that close the eye and those that open it, notably by raising or lowering the eyebrow.

The frontalis is the only muscle innervated by the facial nerve to open the gaze by pulling the eyebrow. The corrugator, procerus, and orbicularis oculi contract to close the eye. It is important to strike a balance between these muscles on the paralyzed side in order to reduce any pre-existing lagophthalmos or, in any case, not to encourage its appearance after the injections. On the healthy side, where there is often hyperactivity of the frontalis, leading to widening of the palpebral slit opening, the doses injected will be higher as the muscle is healthy [8]. There may also be aberrant hyperactivity of the frontalis muscle on the affected side, otherwise known as paradoxical Babinski’s sign, which may be responsible for asymmetry in eyebrow height in favor of the paretic side.

The palpation of these different muscles at rest and during voluntary contraction helps to determine the ideal injection site. The injection plan must be tailored to each patient.

*Frontalis*

*Anatomy and function*

The forehead muscle is vast, superficial, quadrilateral in shape, and intimately adherent to the superficial fascia. Its motor function is to raise the eyebrows and lower the hairline and wrinkle the forehead. The motor plates of this muscle form several independent muscle units. On a horizontal axis, we distinguish between motor plates in the upper part of the muscle, which are responsible for lowering the hairline, and between motor plates in the lower part of the muscle, which are responsible for raising the eyebrows. Some authors divide this muscle vertically into different segments, with motor plates that are equally independent of each other [29].

*Implications for facial paralysis sequelae*

Functional analysis of the forehead, with its height and wrinkles, is essential. Injections can be used to symmetrize the eyebrows and erase wrinkles on the healthy side.

*Injection technique*

Depending on the height of the forehead, the asymmetry observed and the patient’s discomfort/expectations, one-to-eight injections of one-to-two IUs are usually performed on a half forehead. Injections should be superficial, with the needle inserted subcutaneously at one-third of its length so as not to reach the periosteum.

Care must be taken to leave at least two fingerbreadths above the eyebrow without injection to avoid secondary ptosis of the eyebrows and eye on the healthy side.

In the event of a paradoxical Babinski’s sign on the paretic side, and especially if associated with fragile palpebral occlusion, one-to-four injection points of one-to-two IUs each should be made on the side of the sequelae hemi-forehead. We advise not to hesitate to inject into the capillary line to relax the contraction of the upper portion of the frontalis. Injections into the hyperactive area of the muscle are very superficial at 1 IU.

*Corrugator*

*Anatomy and function*

It inserts itself on the medial most part of the eyebrow arch, crossing the muscle fibers of the orbicularis muscle of the eye and terminating in the dermis of the eyebrow skin. It draws the head of the eyebrow downwards, forming vertical wrinkles in the middle of the forehead.

*Implications for facial paralysis sequelae*

Its injection treats vertical medial wrinkles in the glabellar region and corrects asymmetry between the two eyebrow heads. Wrinkles are often more pronounced on the healthy side.

*Injection technique*

Typically, the corrugator requires two-to-three injection points. A superficial injection is made above the outer part of the arch of the eyebrow, which is identified by palpation. The eyebrow should not be used as a reference point as it is not a fixed reference point for the muscle’s cutaneous insertion. A deep injection is performed in the medial third of the eyebrow by palpating the muscle and asking the patient to frown [53].

The injection should be directed upwards, away from the eye socket, to reduce the risk of ptosis by diffusion to the levator muscle of the upper eyelid [54]. The various injection points will help to combat any residual asymmetry at the level of the eyebrow head, as well as any contracture, frequently found at this level in the aftereffects of PFP on the affected side.

*Procerus*

*Anatomy and function*

It inserts onto the outer, lower part of the nasal bone and the upper part of the underlying lateral nasal cartilage. It ascends towards the root of the nose, intertwining its fibers with those of the frontal belly of the occipito-frontal muscle. It ends on the deep surface of the skin at the level of the glabella.

*Implications for facial paralysis sequelae*

Its injection treats asymmetries in horizontal wrinkles at the root of the nose.

*Injection technique*

A deep injection of the interbrow region is performed at two-to-four IUs.

*Orbicularis oculi*

*Anatomy and function*

This a large muscle with concentric fibers arranged around the palpebral fissure. It has three parts: a peripheral orbital part with bony insertions, forming a flat muscular ring around the eyelids and the base of the orbit; a palpebral part with fibrous attachments, covering the entire extent of the eyelid; and a lacrimal pre-tarsal part (posterior lacrimal muscle or Horner’s muscle), which compresses the lacrimal canaliculi and the lacrimal sac.

*Implications for facial paralysis sequelae*

An injection of this muscle treats palpebral asymmetry and periorbicular wrinkles at rest and during contraction. On the healthy side, an injection of the hypertrophied pre-tarsal part of the muscle opens the palpebral fissure, which is often smaller than on the paralyzed side [8].

*Injection technique*

Very superficial injections are made into the orbital part of the muscle at the point where the synkinesis is located, avoiding the medial and upper part of the muscle in relation to the upper eyelid to avoid ptosis [28].

Lagophthalmos and exposure keratitis on the paralyzed side, associated with botulinum toxin injections, are side effects to be avoided [25]. To avoid upsetting the balance between ptosis and lagophthalmos, it is important to know and respect the location and depth of the injection points. Some authors recommend injecting the orbicularis oculi medially into the upper eyelid to avoid ptosis. The patient is asked to look straight ahead to locate the line between the iris and the upper and lower eyelids, and to respect a distance of 5 mm around for the injection. A 45° angle is recommended to avoid diplopia or ptosis caused by accidental injection of the extrinsic or levator muscles of the upper eyelid [25,55].

Injections into the lower eyelid should not be too medial to avoid epiphora or paralytic ectropion. In their initial treatment protocol, Filipo et al. recommend four injections into the orbicularis oculi, with a total dose of five-to-eight IUs around the eye [25].

Borodic et al. show that botulinum toxin injections can increase the vertical palpebral distance and the distance between the upper free edge of the palpebra and the pupil during synkinesis triggered by chewing or smiling. These patients benefit from an improved visual field [8,55]. The balance between the palpebral opening defect and the corneal protection defect is difficult to achieve, as some patients have developed exposure keratitis.

We recommend adapting the number of injection points, their location (pretarsal injections being the most effective, but also the riskiest), the number of units according to the quality of the patient’s palpebral occlusion (look for a cilia sign beforehand), the presence of lagophthalmos, any history of keratitis, and, of course, the degree of orbicular synkinesis. Retro-tracking injections can also be used to act on a greater number of motor plaques since they are uniformly distributed within the muscle [16,56].

*Tear gland*

*Anatomy and function*

An exocrine gland comprises two parts: an orbital portion or main oval lacrimal gland, which is about 20 mm long and responds to the lacrimal gland fossa of the orbital lamina of the frontal bone and a palpebral portion or accessory lacrimal gland, located at the superior lateral part of the upper eyelid. These two portions are separated from each other by a fibro-tendinous plane formed by the external orbital fascicle of the levator muscle of the upper eyelid. It enables the permanent secretion of both tear film and emotional tears.

*Implications for facial paralysis sequelae*

Chronic lacrimation in these patients may be due to excessive tear production, which is associated with crocodile tear syndrome or poor tear drainage due to the paralysis of the orbicular lacrimal pump, palpebral synkinesis, or malposition of the lacrimal point on an ectropion [31,57]. However, the main cause of chronic lacrimation in cases of facial paralysis sequelae is dry syndrome, which should be investigated and treated before considering lacrimal gland injection.

An intraglandular injection of the palpebral part of the lacrimal gland with botulinum toxin provides satisfactory results for chronic lacrimation in these patients [31,58,59,60,61]. Only the palpebral part of the gland should be injected in order to avoid dry eye syndrome.

*Injection technique*

**Figure 1:**
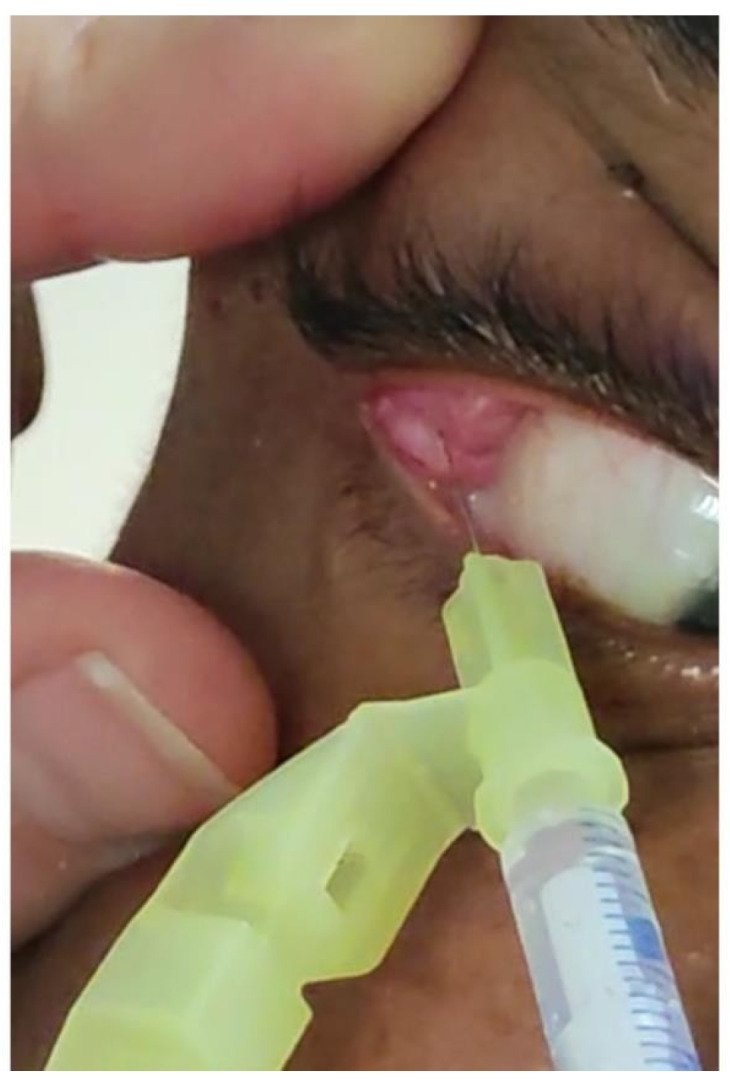
Botulinum toxin injection in the right lacrimal gland.

Classically, we inject one-to-four IUs of botulinum toxin transconjunctivally into the palpebral part of the gland. In the literature, 2-to-10 IUs are injected [62,63,64,65,66,67]. The onset of action and duration of efficacy are different from those of intramuscular injections, with a resolution of epiphora in 1-to-7 days and an average efficacy of 10 months [60]. Ask the patient to look downwards and inwards then highlight the lacrimal gland by lifting the upper eyelid (Figure 1). Uncommon side effects include ptosis, diplopia, or hematoma. To reduce the risk of diffusion, it is preferable to use toxins concentrated in a smaller volume (halved).

#### 4.4.2. Middle Third of Face

Injections of the middle third of the face are more complex than those of the upper third, as residual paresis may persist on the upper lip and zygomatic muscles and a slight overdose can rapidly lead to disabling functional disorders (speech, mastication). An assessment of the muscles involved in cheek and upper lip movement, modulating the smile, is complex. The muscles involved in raising the upper lip are the levator labii superioris, levator labii superior alaeque nasi, levator anguli ori, and minor and major zygomatis. Conversely, the muscles responsible for lowering the lower lip are depressor labii inferiors, depressor anguli oris, mentalis, and platysma.

*Levator labii superiors*

*Anatomy and function*

It inserts itself superiorly at the lower edge of the orbit and laterally to the infraorbital foramen, it runs obliquely downwards and forwards, and it ends by attaching to the deep skin of the upper lip. It elevates the upper lip and the wing of the nose.

*Implications for facial paralysis sequelae*

Its injection corrects the asymmetry of the medial third of the nasolabial fold.

*Injection technique*

The healthy side should be injected if hyperactivity is observed in mimicry, making sure that the upper lip is not lower than the contralateral lip at rest. Typically, one-to-three injections at 1 or 1.25 IUs are performed:

- One superficial on the outer half of the upper lip, 1 cm from the red lip
- A deep one on the outside of the foot of the nostril wing
- A deep paranasal

Perioral injections should be superficial and directed away from the mouth to avoid speech impediments. Some authors recommend not speaking until the day after the injection, as with all perioral injections [30].

*Zygomaticus major*

*Anatomy and function*

This is a cheek muscle that extends from the maxilla downwards and inwards to the corner of the mouth. It lies outside the zygomaticus minor muscle in front of the buccinator muscle. Its contraction raises the angle of the mouth and accentuates the nasolabial fold. It accentuates the curve of the cheekbone, pushing back the soft parts of the cheek.

*Implications for facial paralysis sequelae*

Hyperactivity of the muscle results in an elevation of the oral commissure. Injecting this muscle corrects smile asymmetry on the superolateral, non-paralyzed side.

*Injection technique*

Classically, one-to-two injections of two-to-five IUs are provided:

- One superficial in the nasolabial fold, 0.5 cm from the labial commissure
- A second deep one on the outside, where the maximum number of motor endplates are located, according to Lapatki’s work [17].

*Buccinator*

*Anatomy and function*

This deep cheek muscle is rectangle-shaped and has three fascicles. It inserts into the maxillary bone above and into the mandibular bone and buccinator ridge below. It inserts anteriorly onto the orbicularis muscle of the mouth and posteriorly on the pterygomandibular raphe. It has mobile insertions on the deep surface of the lip, blending with orbicularis muscle fibers. Its function is to keep the cheek pressed against the dental arch, making chewing efficient.

*Implications for facial paralysis sequelae*

Its synkinesis, hypertony, and spasms are common in up to 88% of patients with facial paralysis sequelae. Its identification in muscle areas to be treated with botulinum toxin has only recently been established, in comparison with other muscles classically injected [68,69,70,71,72].

Post-paralytic sequalae damage to these muscles is responsible for jugal pain. Failure to empty the mouth, with food stuck in the vestibule, is a sign of malfunction. Synkinesis can also be responsible for jugal biting and speech difficulties. Using the FGS and SAQ questionnaires, Wei et al. and Patel et al. demonstrated a significant improvement in synkinesis of this muscle with botulinum toxin [70,71,72]. Lacroix et al. developed a specific questionnaire concerning buccinator muscle actions after botulinum toxin injection and showed an improvement in involuntary biting of the inner cheek, difficulty in chewing, disabling drooling, and discomfort in smiling, which they retained as an indication for injection of this muscle [68]. One of the rare and often transient post-injection complications of this muscle is major speech impairment. Its hyperkinesis may be responsible for dental impressions on the jugal mucosa.

*Injection technique*

**Figure 2:**
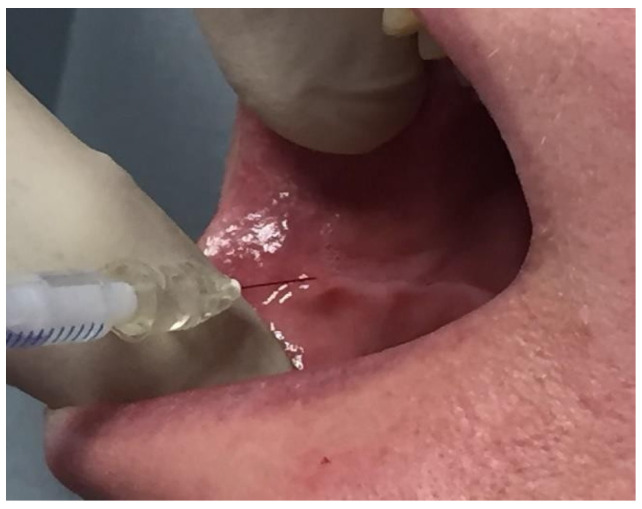
Injection into the buccinator muscle via the endobuccal route after highlighting the dental impression line on the jugal mucosa.

Injection is performed via the endobuccal route, which we recommend for its direct accessibility, precision, and painlessness, or via the external cutaneous route. Endobuccally, the injection is performed perpendicular to the groove marked on the jugal mucosa by the teeth in contact with the buccinator muscle, 1 cm from the buccal commissure (Figure 2). One-to-three injection points of two IUs are typically performed.

#### 4.4.3. Lower Third of Face

*Orbicularis ori*

*Anatomy and function*

It has two parts: a labial part comprising an upper bundle (upper lip) and a lower bundle (lower lip) that attach to the skin at each corner of the mouth, and a marginal part made up of two upper bundles that attach to the maxillary bone opposite the lateral incisor, as well as two lower bundles that attach to the mandible opposite the canine.

All skin muscles acting on the lips converge on this muscle, i.e., from medial to lateral on the upper lip. The muscles that elevate the upper lip and the wing of the nose elevate the angle of the mouth, small zygomaticus, and large zygomaticus, while on the lower lip, the mental muscle lowers it and lowers the angle of the mouth. It constricts the lips and ensures mouth closure. It enables sucking and whistling.

*Implications for facial paralysis sequelae*

Indications for an injection in this muscle are less frequent and concern perioral wrinkles and the correction of a philtrum deviated on the healthy side. Injections must be carefully considered due to the major risk of speech disorders and lip incontinence.

Precautions to limit the spread of the toxin, such as using half doses, steering away from the mouth of the needle, and resting the muscle post-injection, are particularly important. Some authors recommend not injecting within 1-to-1.5 cm of the corner of the mouth and injecting in three sites with a total dose of two-to-six IUs [26].

*Injection technique*

Injections into the hyperactive area of the muscle are very superficial and intradermal at 1 IU.

*Depressor anguli ori*

*Anatomical reminder*

It inserts on the lateral surface of the mandible and terminates on the deep surface of the skin of the angle of the mouth and at the level of the muscle fibers of the orbicularis muscle of the mouth, thus participating in the modiolus [73].

*Implications for facial paralysis sequelae*

Physiologically, the DAO should relax during smiling. Very frequently, the DAO recovers with an aberrant sequelae contraction during smiling, blocking the action of the zygomaticus, to which it is antagonistic, and thus drawing the labial commissure downwards, giving the appearance of a sigmoid smile.

*Injection technique*

**Figure 3:**
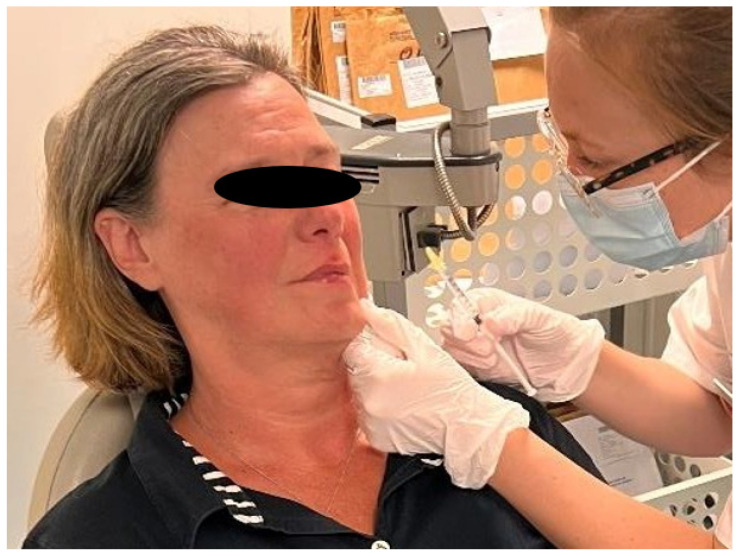
Palpation of the fibrous band of the DAO consistent with its hyperkinesis.

The DAO sign is an important semiological entity to look for. It is represented by the concomitant contraction of the DAO and zygomatic muscles when smiling [30,74]. It is detected by placing the finger on the bitterness fold and asking the patient to smile: a subcutaneous cord is then perceived, corresponding to the abnormal and powerful contraction of the DAO (Figure 3).

The contraction of the DAO causes the labial commissure to lower, as in the expression of disgust. The co-contraction or spasm of this muscle will impede the rise of the labial commissure during smiling, meaning the latter has a sigmoidal shape. Injecting botulinum toxin into this muscle frees up the smile on the paralysed side [75]. It is injected close to the modiolus on the affected side, and lower and next to the mandibular arch, 15 mm from the labial commissure, when the aim is to treat hyperactivity on the healthy side. 

*Injection technique*

It is necessary to treat the two agonist muscles that lower the angle of the mouth: the DAO and the platysma. On the pathological side, one-to-three injection points of 0.5-to-1 IU each are made in the DAO along a line running downwards from the modiolus. The first injection point is located around 1cm outside and 1cm below the labial commissure. The top two injections are subcutaneous, and the lowest injection is deep to reach the deepest part of the DAO (bony insertion).

For the platysma, 4-to-12 points of four-to-five IU botulinum toxin are injected superficially subcutaneously into its medial part, in the downward extension of the DAO. The platysma acts as a functional unit with the DAO, whose motor innervation it may partially share, and, in our experience, it achieves a co-contraction whose treatment is indispensable.

*Mentalis*

*Anatomy and function*

It inserts itself into the lower paramedian part of the chin protuberance and runs obliquely upwards and laterally, ending at the deep face of the chin skin. It raises the chin skin and the lower lip.

*Implications for facial paralysis sequelae*

Synkinesis of the chin muscle is frequently observed in the form of unsightly pads when closing the eyes, smiling, or speaking.

*Injection technique*

One or two deep injections of two IUs in the chin tuft, inthe dimples created, are administered, and several superficial injections to treat the skin insertions of the muscle are also administered [76].

*Platysma*

*Anatomy and function*

This is a large, quadrilateral muscle with a lower cutaneous insertion opposite the acromion and clavicle. It rises obliquely upwards and medially towards the mandible and inserts upwards via both a bony insertion at the lower edge of the mandible and cutaneous insertions at the lower lip and corner of the mouth. It thus lowers the lower lip and labial commissure and tightens the skin of the neck [77].

*Implications for facial paralysis sequelae*

**Figure 4:**
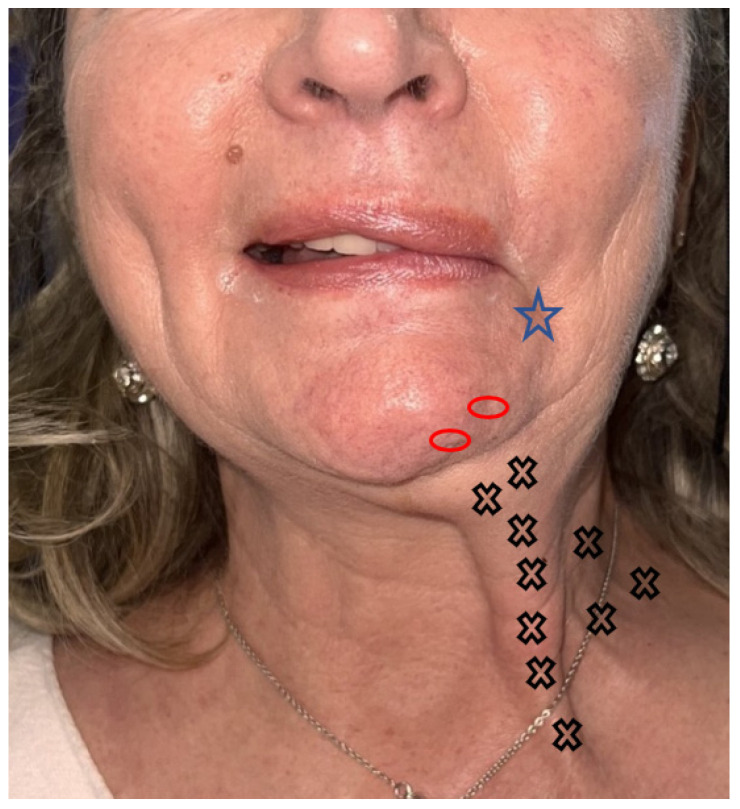
Patient with debilitating hyperkinesis and synkinesia in the mentalis, plastyma and DAO, responsible for asymmetry of the lower third of the face when smiling. Dose and site of botulinum toxin injection protocol suggestion. Black cross (4 UI) for platysma, blue star (2 UI) for DAO, red circle (2 UI) for mentalis.

Synkinesis and contractures are common, affecting up to 63% of patients [78]. They are responsible for neck pain with fibrous cords visible subcutaneously at rest, as well as for a defect in the elevation of the lower lip and labial commissure responsible for asymmetry of the smile (Figure 4) [79]. 

*Injection technique*

**Figure 5:**
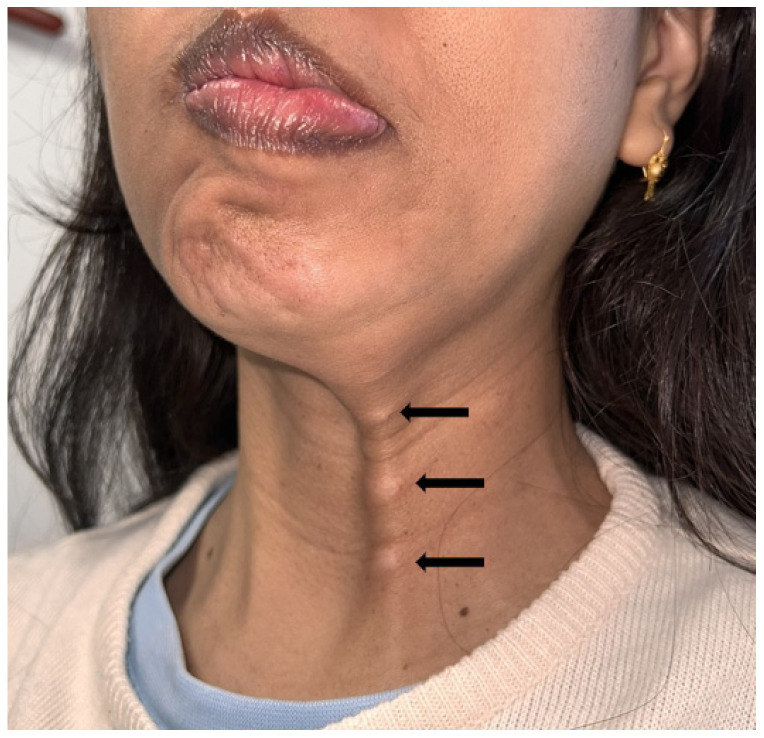
Patient with platysmal band linked to platysmal synkinesis and hyperkinesis. Black arrows showing subdermal injection of four UIs of botulinum toxin.

The topography of this vast superficial muscle requires strict subcutaneous injections along its entire course [78]. Up to 12 injection points of four IUs can cover its surface. There is no need to hesitate to go up along its insertion on the mandibular border. Vertical points (Figure 5) are evenly distributed along the platysmal cords [43].

It may be advisable to dilute the product further to promote diffusion in cases of severe contractures. The risk of the toxin spreading to the larynx is rare, but the needle should be directed away from the larynx and remain strictly superficial [80].

*Other*

In our experience, patients may experience painful contractures of the masseter and sternocleidomastoid muscles on the paralyzed side. These muscles are not innervated by the facial nerve, but disrupted facial balance may induce reactive contractions of these muscles.

Some patients with sequelae of facial paralysis have contractures of the upper sternocleidomastoid. These are responsible for neck and shoulder pain. One-to-three intramuscular injections of five IUs provide effective relief.

Other patients possess masseter contractures on the paralyzed side. Both sides are injected to avoid long-term asymmetry. One injection of five IUs is administered into the muscle on the paralyzed side and two injections of five IUs are administered into the muscle on the healthy side.

### 4.5. Practical Application: Dose

The dose and injection points vary individually according to the severity of synkinesis or hyperkinesy, but also according to the muscle being treated [26,41].

For the first injection session, it is recommended to reduce the injected dose, if necessary, by injecting a further dose 2-to-3 weeks later in order to avoid the undesirable effects of overdosing and to better target the patient’s specific needs. Determining the optimal dose for a patient is empirical. However, the product leaflet’s recommendations of 100 IUs for the face and 300 IUs for the neck should be considered [81].

Shinn et al. reported an average dose of two-to-three units for each facial muscle injected, and 9-to-10 units for the platysma [82]. Choi et al. reported on the paralyzed side, for the treatment of synkinesis, doses of 0.5-to-2.5 IUs per muscle for a total dose of 10-to-26 IUs. On the healthy side, injections into hypertrophied muscles vary from 2.5-to-5 IUs for a total dose of 35-to-72 IUs [9]. For Risoud et al., who studied the distribution and kinetics of doses in 30 patients over 2 years, the average total dose was 20.2 IU ± 11.7, distributed as follows between the two sides: 9.8 IU ± 7.2 for the healthy side, 10.4 IU ± 9.9 for the paralyzed side [5].

In the study by Salles et al., which managed synkinesis in 353 patients, the total dose used per patient ranged from 2-to-106 IUs, with an average of 38 IU +/− 17 [3]. Other authors report higher total doses, ranging from 109-to-156 IUs, in order to treat synkinesis, facial asymmetry spasms, and also to provide patients with a global treatment with an aesthetic component to rejuvenate the face [41,83].

Some authors have developed injection tables for the dose and site [16,83]. Only a standardized assessment of the patient and of the results obtained would allow a comparison of studies reporting on the management of facial paralysis sequelae [42].

**Figure 6:**
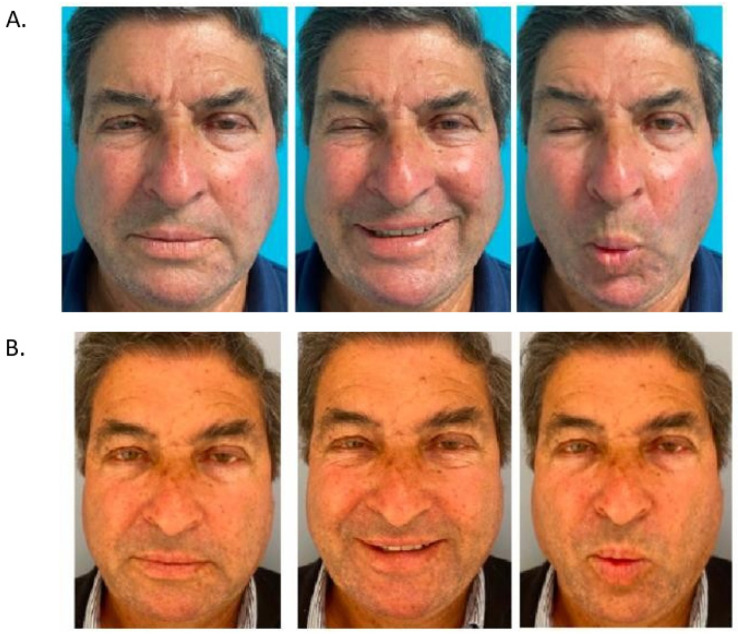
Patient with right mouth–eye hyperkinesis and synkinesis, at rest, smiling, and kissing. (**A**) Before botulinum-toxin injections. (**B**) One month after botulinum-toxin injections.

**Figure 7:**
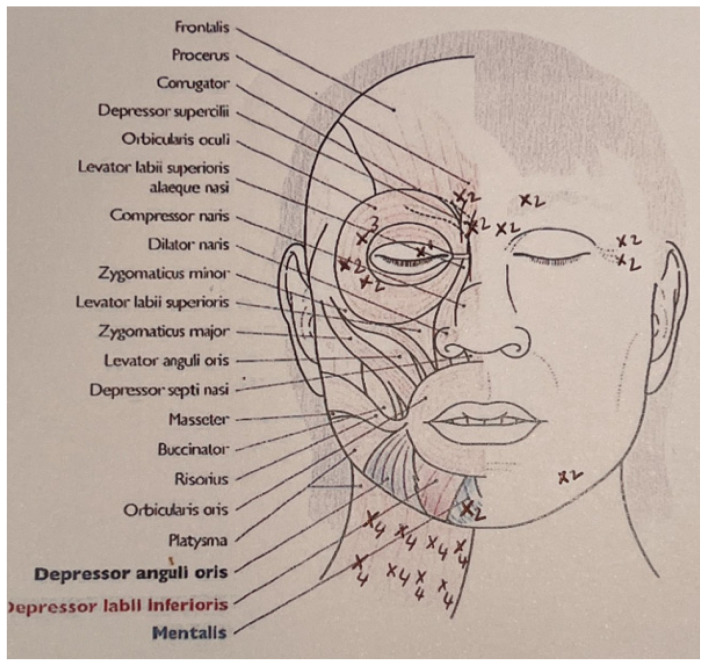
Patient’s treatment plan. Black cross is the injection site, and the number next to it is the number of units of botulinum injection.

In our experience, analysing retrospectively the successive injection records of 88 patients with sequelae of peripheral facial paralysis of any etiology, managed in our center, we studied the number of injections and doses per site [11]. The mean number of injection sites on the paralyzed side was 17.3 ± 5.1, and the mean number of units injected was 44.6 ± 13. Most patients (n = 78; 89%) also received contralateral injections. For the healthy side, the mean number of injection sites was 6.8 ± 3.1, and the mean number of units injected was 13.8 ± 7.6. Figure 6 shows the management of a patient presenting with synkinesia before and after botulinum toxin injection. Figure 7 shows his injection treatment plan.

A recent meta-analysis on 106 patients concluded the treatment of long-term synkinesis is very individual and should focus on a patient-tailored treatment to focus on specific individual complaints instead of trying to standardize treatment [41].

### 4.6. Injection Equipment

Typically, a 1 mL syringe and a 30-gauge hypodermic needle are used for both superficial and deep injections. The use of a pen injector for botulinum toxin enables calibrated, reproducible doses to be delivered without the need for the operator to monitor delivery in real time [84]. The pen injector eliminates the measurement bias associated with the conventional manual-injection procedure. It also makes it easier to perform backtracking injections.

Furthermore, it has been shown that the use of a pen injector in patients with post-paralytic facial hemispasm is a less painful technique than conventional manual-injection procedures [85].

## 5. Follow-Up

### 5.1. Post-Injection Patient Assessment and Precautionary Measures

Botulinum toxin is most effective between the 10th and 30th days after injection. Some authors recommend that a lower dose of botulinum toxin be administered initially, with an additional second injection 15 days later to minimize the risk of side effects in certain locations and achieve optimal facial symmetrization [3,27].

Once the patient’s response to botulinum toxin at each injection site is known, it can be ideally adjusted and maintained over the course of several sessions. Shinn et al. report the need for three injection sessions to determine the optimal dose of botulinum toxin per muscle group [54,82]. Careful monitoring of the patient and his or her response to the toxin after each injection session helps to achieve better results and to adjust doses and injection points in good time.

As with cosmetic botulinum-toxin injections, patients are advised not to massage their face and the injected areas for up to 1 week after the injections to avoid diffusion of the toxin to adjacent muscles [12,50]. After frontal-muscle injections, it is not advisable to wear a motorcycle helmet or bathing cap. After perioral injections, excessive talking should be avoided for 24 h. You should also avoid hot areas such as saunas and steam baths for 5 days following toxin injections to avoid inactivating the toxin.

### 5.2. Injection Rate

Although the patient’s opinion is consulted, the muscles selected, the dose applied, and the frequency of injections are a medical decision that must consider the clinical presentation of facial paralysis and the results of previous injections in particular.

The frequency of injections required to treat the aftereffects of peripheral facial paralysis varies from 3-to-4 months on average, depending on the patient, when they feel that botulinum toxin is no longer effective against hyperkinesis and synkinesis [86,87]. The time between injections is stable in the time. In some centers, where the large volume of patients under care means that such close monitoring is not possible, this time may be extended. Hernandez Herrero et al. routinely injected patients every 4 months [87]. Other authors choose to inject patients every 5 months [88] or every 6 months [89]. In our opinion, it is necessary to wait for complete resolution of the toxin’s efficacy in order to avoid anarchic reappearance of sequelae in the weeks following injection.

### 5.3. Long-Term Use and Resistance to Botulinum Toxin

Alipour et al., in a series of injections involving 73 patients over 20 years, observed an increase in the number of muscles injected, even if the doses remained the same [86]. Shinn et al. report an average of six muscles injected, whatever the etiology of facial paralysis [82]. Salles et al. report a progressively increasing average dose over time [3]. No clinical resistance to the toxin was observed over their 11-year follow-up. In the Risoud et al. series, dosage kinetics suggest an initial increase in dose after the first session, followed by a decrease over time [5]. Whatever the practice, the orbicularis oculi, mentalis, DAO, and platysma muscles are the most frequently injected [79,86].

Methods of use and doses are variable due to the dynamic evolution of syncinesis and spasms, the multitude of facial muscles involved, the variation reported by the patient in the muscles involved in his discomfort, and the preferences of the injector. Some centers report the systematic treatment of predefined muscles: orbicularis oculi, risorius, mentalis, platysma, and corrugator [54].

Resistance to the botulinum toxin is rare but can appear over time. Classically, a predisposition to failure is demonstrated in these patients, with the need to require increasing doses of toxin in the face of a reduced muscle response to its effect during successive injections [82]. The mechanisms of resistance have not been elucidated but are thought to be secondary to an immune response. Risk factors are the administration of increasing doses of botulinum toxin, an increase in the frequency of injections, and a large number of injections over lifetime. In such cases, the toxin formulation or subtype should be changed.

### 5.4. Undesirable Effects and Complications

Undesirable effects are most often linked to diffusion of the toxin beyond the targeted muscle. They may be related to too high a dose, an unsuitable conversion ratio, too large an injection volume, or technical errors. Poor compliance with post-injection recommendations can also be the cause (massage, make-up removal, sport, swimming, etc.).

In the mid and lower face, the most frequent adverse effects are slurred speech or mastication [26,40]. They diminish over time as the effect of the botulinum toxin diminishes and patients adapt to their new facial dynamics [8]. Hematomas at the injection site are uncommon and of no functional consequence.

In the upper face, especially in the periorbital region, the main complications secondary to botulinum toxin injections are the ptosis of the eyelid or eyebrow, eyebrow asymmetry, diplopia, lagophthalmos, palpebral ectropion, and the prominence of the inferior palpebral bags [58,59,90].

Lagophthalmos can occur when the injected dose in the orbicularis oculi muscle is too high, which is the leading cause of a lack of eye occlusion and xerophtalmia. It should be treated with eye drops and lubricating gels, as well as nocturnal palpebral closing [90]. Sometimes, injections of the frontalis in its lower part allow for a ptosis improvement as this muscle antagonises the orbicularis oculi [50]. Blepharoptosis can occur when a large dose of botulinum toxin is provided, or when the injection site is close to the orbital rim where the supraorbital neurovascular pedicle can be a shortcut, or when the injection is not the dilution is not concentrated and the product diffuses [57]. It is responsible for the paralysis of the levator palpebrae superioris muscle. This complication self-resolves at the end of the botulinum toxin action. Oxymetazoline hydrochloride or apraclonidine hydrochloride eye drops, anticholinesterase agents, partially reverse this adverse effect. An intradermal injection of concentrated botulinum toxin in the pre-tarsal orbicularis can also be useful to decrease the ptosis [57,90]. The anatomic study shows that a supraorbital foramen may be present in some patients and constitutes a shortcut from the brow area directly into the orbital roof, following the supraorbital neurovascular pedicle.

Diplopia, which is a rare complication, may result from an unintended paresis of the rectus inferioris or lateralis. When it occurs, the medial rectus becomes hyperactive, causing the pupil to shift to the medial side. The patient has to wait for the effect of the toxin to wear off [12,90].

Brow asymmetry, or samurai eyebrows, can be managed by injecting into a suitable area of the frontalis to balance the face [58].

A perfect knowledge of the periorbital anatomy allows for the avoidance of the danger zone and for the upper face to symmetrize it when needed.

## 6. Conclusions

Botulinum-toxin injections, a key tool in the management of the aftereffects of facial paralysis, improve patients’ quality of life and functional impotence. It is a balancing act that requires experience and expertise on muscles that are both paretic and spastic.

A good knowledge of botulinum-toxin characteristics and anatomy is essential to offer patients an effective and safe injection protocol. A careful assessment of the patient and his or her complaints prior to injection is the key to optimal treatment tailored to each individual patient.

## Figures and Tables

**Table 1:** Different types of toxins and equivalences associated with recommended dose and dilution.

Toxin Type	Dose per Injection	Dilution
Botox	2–2.5 IU	1 to 1.25 mL for 50 IU
Xeomin	2–2.5 IU	1 to 1.25 mL for 50 IU
Dysport	20–25 IU	2 to 2.5 mL for 500 IU

Several factors influence the diffusion of the toxin once injected: preparation characteristics, volume, dosage, injection technique, and injected muscle. Dilution is modulated on a case-by-case basis. It may be useful to increase the concentration of toxins in order to reduce the phenomenon of diffusion, particularly for high-risk areas and small muscles. On the other hand, a higher dilution can be used on larger muscles, notably the platysma.

## Data Availability

No new data were created or analyzed in this study. Data sharing is not applicable to this article.
